# Functional Diversity and CO_2_ Emission Characteristics of Soil Bacteria during the Succession of Halophyte Vegetation in the Yellow River Delta

**DOI:** 10.3390/ijerph191912919

**Published:** 2022-10-09

**Authors:** Yu Xin, Linhui Ji, Zihao Wang, Kun Li, Xiaoya Xu, Dufa Guo

**Affiliations:** College of Geography and Environment, Shandong Normal University, Jinan 250014, China

**Keywords:** greenhouse gas, microorganisms, vegetation succession, high-throughput sequencing, wetlands

## Abstract

Carbon dioxide (CO_2_) is the most important greenhouse gas in the atmosphere, which is mainly derived from microbial respiration in soil. Soil bacteria are an important part of the soil ecosystem and play an important role in the process of plant growth, mineralization, and decomposition of organic matter. In this paper, we discuss a laboratory incubation experiment that we conducted to investigate the CO_2_ emissions and the underlying bacterial communities under the natural succession of halophyte vegetation in the Yellow River Delta by using high-throughput sequencing technology and PICRUSt functional prediction. The results showed that the bacterial abundance and diversity increased significantly along with the succession of halophyte vegetation. Metabolic function is the dominant function of soil bacteria in the study area. With the succession of halophyte vegetation, the rate of CO_2_ emissions gradually increased, and were significantly higher in soil covered with vegetation than that of the bare land without vegetation coverage. These results helped to better understand the relationships of soil bacterial communities under the background of halophyte vegetation succession, which can help to make efficient strategies to mitigate CO_2_ emissions and enhance carbon sequestration.

## 1. Introduction

Global warming is a serious environmental issue that the world is currently dealing with [1], and it is primarily caused by an increase in greenhouse gases such as carbon dioxide (CO_2_), methane (CH_4_), and nitrous oxide (N_2_O) in the atmosphere [2]. According to the IPCC (2021), CO_2_ is the most significant greenhouse gas, and an increase in its concentration causes the greenhouse effect, which is the primary contributor to climate change. CO_2_ contributes up to 70% to climate change issues [3]. Wetlands are ecosystems formed by the interaction of water and land, which can absorb or release greenhouse gases such as CO_2_ [4]. With only 5% of the land area [5], wetlands hold up to 770 Gt of carbon, far more than forest ecosystems and agricultural ecosystems, making them the largest carbon pool in terrestrial ecosystems. The pathways of wetland CO_2_ emissions mainly include soil respiration and microbial decomposition of organic matter [6], which is the integrated product of soil biometabolism and biochemical processes [7]. Soil respiration is the process of CO_2_ production by the metabolism of plant roots, soil animals, and microorganisms in the soil [8], and its intensity can represent the available nutrient status and organic matter mineralization rate of the soil, which is a good indicator of soil microbial activity and evaluation of soil fertility status [9]. Soil respiration releases a tremendous amount of CO_2_, which is 13 times more than that produced by burning fossil fuels [10], making this process an important part of the carbon cycle in terrestrial ecosystems as well as the main pathway for carbon release from soil carbon pools to the atmosphere [6].

Microorganisms play a highly essential role in soil ecosystems, not only participating in the formation and development of soils [11], but also playing an irreplaceable role in the cycling of various elements on Earth, especially in the cycling of carbon and nitrogen [12]. Through decomposition and mineralization, microorganisms are key drivers of CO_2_ release to the atmosphere through the conversion of soil organic matter to CO_2_ [13]. CO_2_ emissions would be affected by various factors such as soil temperature [14], moisture [15], pH [16], nitrogen [17], and soil texture [10]. Environmental factors indirectly regulate the soil carbon cycle process by affecting the abundance and activity of microorganisms, and the high sensitivity of microorganisms to environmental changes makes them good indicators of environmental threats [18]. Bacteria account for up to 90% of the total number of soil microorganisms [19], and play a very important role in mineralization, decomposing organic matter, and promoting the energy cycle of soil materials [20,21]. Changes in their abundance, community structure, and function would have a significant impact on soil quality and global climate [22], which makes them an ideal indicator of environmental changes. Microbial groups with similar metabolic functions are the basic functional units that constitute the microbial community [22]. Each unit jointly drives the soil material energy cycle, such as organic matter decomposition, energy metabolism, nitrification, and denitrification. The abundance of soil functional genes can be used to represent the potential of soil element cycling [23]. The change of vegetation type will change the above-mentioned environmental factors [24], thus affecting the survival and reproduction of microorganisms, thus having an impact on the soil’s CO_2_ emission fluxes. Liu Shi [25] found that the CO_2_ emissions from broad-leaved forest soil were higher than those of a coniferous forest. Cao [26] conducted a laboratory simulation study on the greenhouse gas emissions from salt marsh soils with different vegetation types in the Ulansu lakeside zone and found that soil moisture and total carbon were the main factors affecting the CO_2_ emissions of the wetland, and that the *Phragmites australis* marsh has the largest CO_2_ emissions. On the northeastern Tibetan Plateau, Chen et al. studied the relationship between soil respiration and microbial communities following afforestation. They discovered that a change from an Actinobacteria-dominated community to an Alphaproteobacteria-dominated community may also be a factor in the rise in soil respiration [27]. In a study on the CO_2_ emission characteristics under various plant communities on the shores of the saline lake in an arid environment, Li discovered that, in addition to soil temperature, salinity is also a significant factor impacting soil carbon emissions along the salt lake [28].

The Yellow River Delta is a new land formed by the deposition of sediment carried by the Yellow River to the mouth of the sea [29]. It is a natural reserve with the fastest natural growth in land area around the world [30]. The Yellow River Delta has great potential for development and utilization, however, due to the periodic submersion under seawater and strong evaporation in the local area [31], the soil salinization phenomenon is serious and the ecological environment is very fragile. The difference in soil desalination degree is caused by the difference in distance from the sea and the time of terrestrial formation. This forms a series of vegetation successions with different degrees of salt tolerance, including highly, moderate, and light, starting from bare land [31], which is a natural laboratory for studying the relationship between microbial communities and vegetation succession in saline soil environments. Soil salinization is a major environmental problem affecting sustainable development [15]. Soil salinity not only has a direct impact on plant growth [32], but also reduces soil respiration rate [33], which weakens microbial activity and the ability to decompose organic matter and in turn leads to a decrease in soil quality [34]. Therefore, understanding the distribution of microorganisms in saline soil and improving the survival environment of microorganisms are of great significance for sustainable development. In recent years, research on soil microorganisms and CO_2_ emissions mainly focused on the differences and influencing factors of microbial abundance and community structure under different vegetation types [35,36,37] and characteristics of soil CO_2_ emissions from inland ecosystems such as forests [16], grasslands [38], and cropland ecosystems [39]. Wetland ecosystems are rarely studied in the literature [40], particularly the functional changes of soil microorganisms and CO_2_ emissions in different vegetation types in the Yellow River Delta wetlands. Vegetation types could not only change the structure of soil microbial communities, but also have a profound impact on the function of terrestrial ecosystems and landscapes [41]. Soil microorganisms have a sensitive response to vegetation succession and environmental changes, which can cause changes in microorganisms’ respiration and in turn have an impact on CO_2_ emissions. Therefore, research on bacterial functional structure diversity and CO_2_ emissions along with the succession of halophyte vegetation in the Yellow River Delta are needed.

In this study, bacterial 16s rRNA high-throughput sequencing and PICRUSt function prediction analysis were used to explore the responses of bacterial community structure, functional diversity, and CO_2_ emissions in the Yellow River Delta along with the halophyte vegetation succession. We hypothesized that (1) the bacterial community structure and functional structure would change along with the halophyte vegetation succession process; (2) the functional abundance of soil bacteria would be closely related to soil CO_2_ emissions; (3) salinity is likely to be the key factor for the changes in the bacterial community and soil CO_2_ emissions during the vegetation succession process.

## 2. Materials and Methods

### 2.1. Study Site and Soil Sampling

The study area is located in the Yellow River Delta of Dongying City (37°26′16.7″ N–37°32′41.4″ N, 118°44′14.1″ E–118°55′10.3″ E), which is a warm temperate monsoon climate zone, with an average annual temperature of about 13 °C, and flat terrain. The Yellow River Delta is formed by alluvial loess carried by the Yellow River and the soil parent materials are Yellow River alluvium, while the soils are Solonchaks and Cambisols (WRB: World Reference Base for Soil Resources). The vegetation types in this area are simple in structure, and the sequence of vegetation succession is bare land (BL)—*Suaeda salsa* (L.) Pall. (SS)—*Tamarix chinensis* Lour. (TC)—*Aeluropus sinensis* (Debeaux) Tzvel. (AS)—*Imperata cylindrica* (L.) Beauv. (IC)—*Artemisia capillaris* Thunb. (AC). The bare land and the soil of highly salt-tolerant vegetation (SS, TC) belong to Solonchaks, while the soils of moderate salt-tolerant vegetation (AS) and lightly salt-tolerant vegetation (IC, AC) belong to Cambisols.

**Figure 1:**
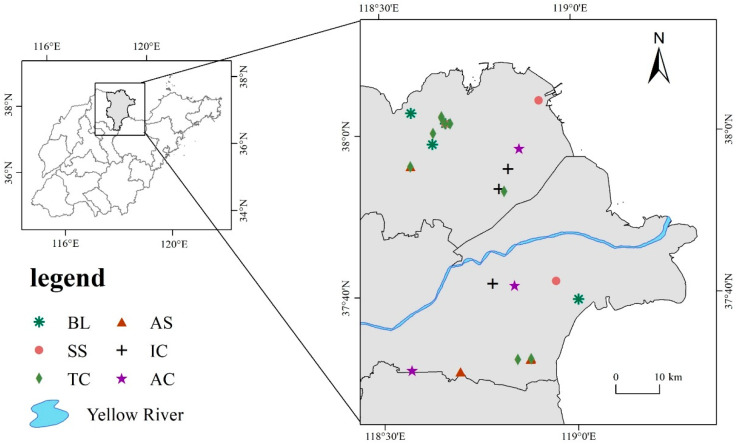
Sampling site in the Yellow River Delta; BL stands for the soil of bare land, SS stands for the soil of *Suaeda salsa* (L.) Pall., TC stands for the soil of *Tamarix chinensis* Lour., AS stands for the soil of *Aeluropus sinensis* (Debeaux) Tzvel., IC stands for the soil of *Imperata cylindrica* (L.) Beauv., and AC stands for the soil of *Artemisia capillaris* Thunb., the same is represented below.

Through multiple field surveys in the Yellow River Delta, and considering local soil salinity, vegetation growth, and distribution, and according to the sequence of succession of halophyte vegetation in the Yellow River Delta, *Suaeda salsa* (L.) Pall., *Tamarix chinensis* Lour., *Aeluropus sinensis* (Debeaux) Tzvel., *Imperata cylindrica* (L.) Beauv., *Artemisia capillaris* Thunb. soil and bare land soil were sampled and located by GPS. These six plots were used to represent the natural succession process of halophyte vegetation in the study area. Sample plots were spaced at least 1 km apart, and three sampling sites were selected for each plot, with three sets of replicates were taken per sampling site. A total of 18 sample plots were selected (Figure 1). Soil layers from 0–20 cm were sampled and mixed according to the five-point sampling method [42]. Plants and gravel were removed from the collected soil samples, soils were sieved through 2 mm meshes, divided into three parts, and placed in sterile sealed bags. One part was stored in a refrigerator at −80 °C for soil DNA extraction, another part was stored in a refrigerator at 4 °C for CO_2_ emissions determination in soil laboratory incubation experiments, while the rest of the soil was naturally air-dried for soil physical and chemical property determination.

### 2.2. Determination of Soil Environmental Factors

The soil temperature was measured with a portable plug-in geothermometer. Soil conductivity was measured with a conductivity meter (DDS-307A, Shanghai, China) to characterize soil salinity [43]. Soil pH [43] was measured with a pH meter (pH-307A, Shanghai, China), and soil organic matter and total nitrogen were determined by an elemental analyzer [44] (Vario MACRO CUBE, Frankfurt, Germany). Soil moisture content was determined by dry weighing at 105 °C [44]. The sodium bicarbonate leaching-molybdenum-antimony resistance colorimetric method was used to determine a soil’s available phosphorus [45]. The alkaline hydrolysis diffusion method was used to determine soil alkali-hydrolyzable nitrogen [46]. A flow analyzer (SEAL Analytical Gmbh AA3) was used to measure soil ammonium and nitrate nitrogen content [47].

### 2.3. Laboratory Experiments for CO_2_ Measurement

The rate of soil CO_2_ emissions was evaluated using a laboratory incubation method to explore the impact of soil microorganisms on CO_2_ emissions and to avoid the complex function of other factors like roots. The fresh soil equivalent to 60 g of dry soil was weighed into a 250 mL culture bottle and pre-incubated at 25 °C for 7 days to activate soil microorganisms. Taking into account that coastal areas in the Yellow River Delta are often inundated with seawater, the soil water content was adjusted with deionized water at a ratio of 1:1 of water to soil during formal culture. This was followed by unsealed incubation at 25 °C in the dark for a total of 21 days [48]. During the culture period, the water in the bottle was supplemented by a weighing method, and the CO_2_ concentration was measured on days 1, 2, 3, 4, 7, 10, 13, 16, and 21. The bottle mouth was sealed with a flip stopper when collecting gas, gas was pushed in the bottle three times with a 10 mL syringe to ensure that the gas is evenly mixed, a 5 mL gas sample was drawn, its concentration was measured with an Agilent gas chromatograph, and then extracted with a syringe. We added 5 mL of air to the culture bottle, kept the pressure in the bottle consistent, and after 40 min, measured the concentration of the gas sample again, we then used the concentration difference between the two measurements to calculate the CO_2_ emission rate on that day.

Emission rate: F(μg/(kg·d)=ΔC×V(mL)×44×273/(273+T)×h×m×22.4

where ΔC is the gas concentration change value per unit of time (μg·L^−1^), V is the volume of gas in the flask, 44 is the Molar mass of CO_2_, T is the incubation temperature (25 °C), h is the closed incubate time (h), m is the dry soil weight (60 g), and 22.4 is the molar volume of CO_2_ at absolute zero (273 K) [49].

### 2.4. DNA Extraction and High-Throughput Sequencing

Total DNA from soil samples was extracted using a Fast DNA Kit (MP Biomedicals, USA). The bacterial V3-V4 region was amplified with bacterial universal primers 515F (GTGCCAGCMGCCGCGGTAA) and 806R (GGACTACHVGGGTWTCTAAT) [50]. The PCR amplification products were purified and recovered using a Gene JET gel recovery kit (Thermo Scientific, USA). The library was constructed by Beijing Novogene Technology Co., Ltd. and the amplification products were sequenced using the ThermofisherIon S5^TM^XL sequencing platform. After the OUT abundance was normalized, the bacterial sequencing results were predicted by PICRUSt software and compared with the KEGG database.

### 2.5. Data Analyses

One-way ANOVA and multiple comparisons were performed using SPSS 21.0 software to compare the significant differences in soil physicochemical factors among the plots. Mantel analysis, SIMPER analysis, and calculation of soil bacterial diversity were performed with PAST software. At the National Microbial Science Data Center, LEfSe analysis of the bacterial community structure was performed, and community evolutionary branching was mapped. The PICRUSt software was used to predict the function of soil bacteria. A correlation analysis of the abundance of predicted functional pathways in soil bacteria was performed and significance tests were performed. Functional abundances with significant differences in the 2-level functional classification of soil bacteria in different soils were compared using the Tukey-Kramer method of STAMP software. A principal function RDA analysis of bacterial level 2 functional classification was plotted using the Canoco 5 software and the effects of soil environmental factors as well as soil predicted functional genes on CO_2_ emission rates were analyzed using partial least squares method in R language.

## 3. Results

### 3.1. Soil Chemical Factors

The soil physicochemical properties of the various plots in the study area are shown in Table 1. Soil salinity ranged from 0.24 dS m^−1^ to 4.83 dS m^−1^, with a gradient of variation across the region. Among them, the lightly salt-tolerant vegetation (IC and AC) has the lowest soil salt content, followed by the moderate salt-tolerant vegetation (AS) and the highly salt-tolerant vegetation (SS, TC), and the bare land has the highest salt content. The succession process of halophyte vegetation has an important influence on a soil’s physical and chemical properties. The soil total nitrogen, organic matter, available phosphorus, alkali-hydrolyzable nitrogen, and ammonium nitrogen contents in the vegetation-covered plots were higher than those in the bare land. Soil total nitrogen, organic matter, alkali-hydrolyzable nitrogen, and ammonium nitrogen were all the highest in AS land, with levels of 0.57 g·kg^−1^, 17.06 g·kg^−1^, 43.58 mg·kg^−1^, and 41.17 mg·kg^−1^, respectively. Soil organic matter, available phosphorus, alkali-hydrolyzable nitrogen, and ammonium nitrogen were the lowest in bare land, and their contents were 5.87 g·kg^−1^, 2.24 mg·kg^−1^, 12.30 mg·kg^−1^, and 1.09 mg·kg^−1^, respectively. There was little difference in available phosphorus and nitrate nitrogen among the six plots, and their contents were between 2.24–3.86 mg·kg^−1^ and 2.25–3.71 mg·kg^−1^. In summary, with the regular changes of salinity, the physicochemical factors of the soil differed significantly, forming a certain ecological gradient, with the soil nutrient content being highest in moderate salt-tolerant vegetation soil, followed by lightly salt-tolerant vegetation soil, and lowest in highly salt-tolerant vegetation soil and bare land.

### 3.2. The Composition of Soil Bacterial Communities

Table 2 shows the alpha-diversity index of soil bacterial communities in various plots during the succession of halophyte vegetation in the Yellow River Delta. The bacterial coverage of all six plots was greater than 96%, indicating that the sequencing depth was reasonable and could represent the real situation of the samples. The number of OTUs of the bacterial communities increased during the succession of halophytic vegetation. The Shannon, Simpson, Chao1, and ACE indices of the bacterial community in the bare land were significantly lower than those in the soil covered with vegetation, indicating that the bare ground had the lowest bacterial diversity and abundance, followed by SS < TC < AS < IC < AC. In conclusion, the OTU number of soil bacterial communities increased with the succession of saline vegetation, with the highest bacterial diversity in soils having mildly salt-tolerant vegetation. Furthermore, a Mantel test showed that soil salinity was significantly correlated with the bacterial community diversity index (R = 0.53, *p* < 0.01).

Bacterial clades with relative soil abundance greater than 1% were selected for analysis, while those with relative abundance less than 1% were combined into another bacterial clade (Figure S1). The main bacterial phyla in the soil of the study area were Proteobacteria, Bacteroidetes, Actinobacteria, Firmicutes, Acidobacteria, Gemmatimonadetes, Chloroflexi, and Planctomycetes. Among them, Proteobacteria had the highest abundance, accounting for 48.54–78.29% of the total bacterial abundance and were the dominant bacterial phylum in the study area. With the succession of halophyte vegetation, the abundance of Proteobacteria continued to decline. Bacteroidetes and Firmicutes had the highest abundance in the soil of TC, while Actinobacteria and Acidobacteria showed a trend of increasing relative abundance with the succession of halophyte vegetation.

### 3.3. Differences in the Distribution of Soil Bacterial Communities

UPGMA cluster analysis showed that soil bacterial communities of different salt-tolerant vegetation had significantly different spatial distribution patterns (Figure S2a). Bacterial communities of soils with the same salt-tolerant vegetation type clustered together as a single cluster, while bacterial communities of soils with different salt-tolerant vegetation types were farther apart, which indicated that bacterial community structures were obviously similar at the same salinity level, while bacterial communities of soils with different salt-tolerant vegetation were far apart. In addition, to identify the key species that caused the differences in bacterial community structure, LEfSe analysis was performed on soil bacteria in the study area at all taxonomic levels (LDA threshold was set to 4), and a community evolutionary branching diagram was constructed (Figure S2b). Circles represent the phylum, class, order, family, and genus of the bacterial community and nodes represent bacterial species, the larger the node, the higher the average relative abundance. Yellow nodes represent bacterial species with no significant difference between groups, and other colored nodes represent significantly different bacterial species. The letters in the Figure S2b correspond to the legend on the right, indicating the taxonomic name of the species.

The results showed that there were significant differences in the bacterial community composition of different vegetation types in the Yellow River Delta. A total of 53 differential indicator species were found in six sample sites, including 11 in bare land, 7 in SS soil, 9 in TC soil, 6 in AS soil, 7 in IC soil, and 13 in AC soil. At the level of phylum classification, Proteobacteria in bare land, Bacteroidetes in TC soil, Chlorobacterium in AS soil, Acidobacteriaceae in IC soil, and Actinobacteria in AC soil are the dominant bacteria phylum in soils of different halophyte vegetation types. At the taxonomic level of genera, *Stenotrophomonas* and *Pseudomonas* in bare land, *Marinobacterium* in AS soil, *Marinobacter* in the SS soil, *Lysobacter* in the IC soil, and *Sphingomonas* in the AC soil are the dominant genus in the soils of various halophytes soil, and no significant dominant genes were found in the soil of TC with significant advantage. SIMPER analysis further identified key species that contribute to differences in soil bacterial community structure during the succession of halophytic vegetation in the Yellow River Delta. The Supplementary Materials Table S1 lists the contribution rates of major bacterial groups to spatial dissimilarity (contribution rate >2%). In general, *Stenotrophomonas* was the bacterial genus that contributed the most to the difference in soil bacterial community structure during the succession of halophytic vegetation in the study area.

### 3.4. Predictive Functional Characteristics of Soil Bacteria during the Succession of Halophyte Vegetation

At present, relatively few studies have been conducted on the ecological function of wetland soil bacteria, while the fundamental pathway for soil CO_2_ emission is heterotrophic respiration by soil microorganisms, which leads to their unique functions [51], making functional gene abundance a good indicator to quantify the functions of soil microorganisms. Findings combination of functional gene abundance with various environmental factors and soil CO_2_ emissions can indicate the ecological functions of soil microorganisms [52]. In this study, PICRUSt 2 software was used to predict the function of soil bacteria, and then the prediction results were compared with the KEGG database. In addition to the unclassified functional groups, a total of six categories of metabolic pathways were annotated in the 1-level predicted functional layer (Figure S3), with the succession of halophyte vegetation, the abundance of the six types of metabolic pathways gradually increased, and metabolism, genetic information processing, environmental information processing, and cellular processes accounted for more than 80% of the 1-level predicted functional layer. The largest abundance was the metabolism pathway, which was the most important function of the soils in the study area. Human disease and organismal systems were also included, and these two metabolic pathways were relatively less abundant and were not the main functions of soil bacteria in the study area. 40 sub-functions were annotated in the level 2 predicted function layer, which reflected the diversity of bacterial functions.

**Figure 2:**
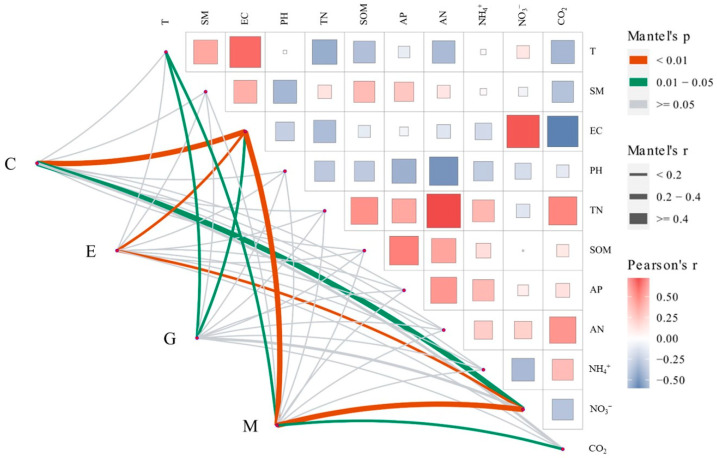
Correlation between 1-level functional prediction classification and environmental factors of soil bacteria in the Yellow River Delta; M: metabolism; G: genetic information processing. E: environmental information processing; C: cellular processes. Notes: The correlation coefficients between environmental factors are represented by the area of the square, the larger the area, the larger the correlation coefficient, blue indicates a positive correlation, red indicates a negative correlation, and the degree of correlation between bacterial function and environmental factors is represented by the width of the line, Different line widths indicate different R-values after the mantel test, the wider the line the stronger the correlation.

By performing the Mantel test on the primary metabolic pathways of bacteria and soil environmental factors (Figure 2), the results showed that electrical conductivity (salinity) was significantly negatively correlated with the four major predicted functional pathways of metabolism, genetic information processing, environmental information processing, and cellular processes, indicating that salinity significantly inhibited microbial activity. Both metabolism and genetic information processing were significantly correlated with temperature, but the temperature was significantly negatively correlated with metabolism, and significantly positively correlated with genetic information processing. Metabolism and environmental information processing, as well as cellular processes, were also significantly correlated with nitrate nitrogen content which indicates that soil nitrogen content would significantly affect the physiological activities of microorganisms. It also demonstrates that only metabolism gene abundance was significantly correlated with soil CO_2_ emission rate, indicating that soil microbial activity in the study area was mainly based on the metabolism of various substances.

The relative abundance of the sub-functions of the 2-level predicted functional layer greater than 1% is listed in the Table S2 and Figure S4, with a total of 22 sub-functions. The abundance of genes predicted by 2-level functions also increased with the succession process of halophyte vegetation, which is consistent with 1-level function. Metabolism is the most important type of functional pathway, and its pathway contains 12 secondary pathways. Their relative abundances are shown in the Figure S4. Metabolism of various substances in the study area occupies most of the functions in the 2-level predicted functional layer. The five functions of amino acid metabolism (10.80%), membrane transport (10.07%), carbohydrate metabolism (9.59%), replication and repair (7.26%), and energy metabolism (5.76%) are the main sub-functions of the soils in the study area (relative abundance >5%).

**Figure 3:**
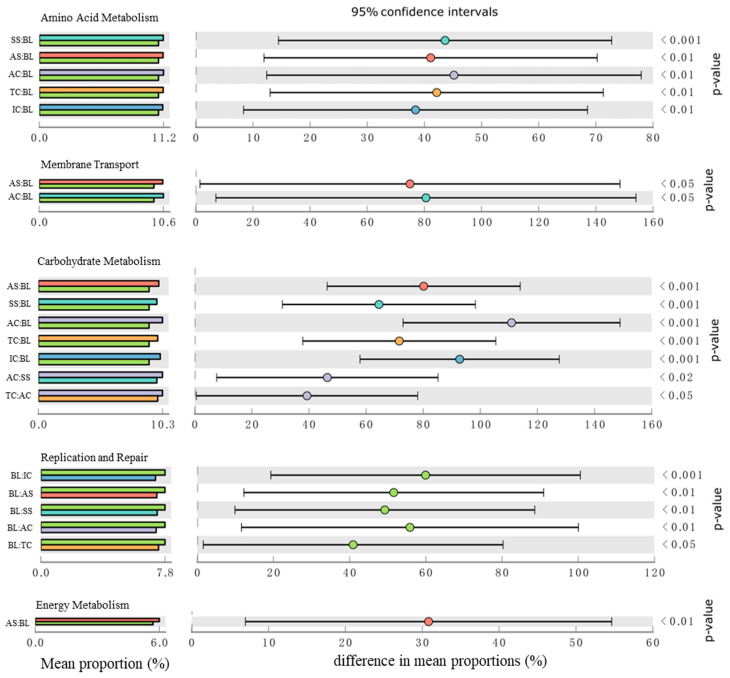
Significant difference analysis in soil bacterial function between plots during the succession of halophyte vegetation in the Yellow River Delta.

Significant differences were analyzed for five major sub-functions of the 2-level predicted functional layer (Figure 3) and the results showed that the functional genes of soil bacteria in the study area showed significant differences with the succession process of halophyte vegetation. Soil bacteria can evolve a variety of physiological adaptation mechanisms to cope with changes in their survival environment. Vegetation-covered soil has more genes related to amino acid and carbohydrate metabolism than bare land, and in the process of halophytic vegetation succession, compared with the highly salt-tolerant vegetation, the lightly salt-tolerant vegetation type soil had higher abundances of carbohydrate metabolism-related genes. In terms of energy metabolism-related genes, the abundance of moderately salt-tolerant vegetation AS and lightly salt-tolerant vegetation AC was significantly higher than that of bare ground, while the abundance of replication and repair-related genes was highest in bare ground and significantly higher than that of vegetated cover soils.

**Figure 4:**
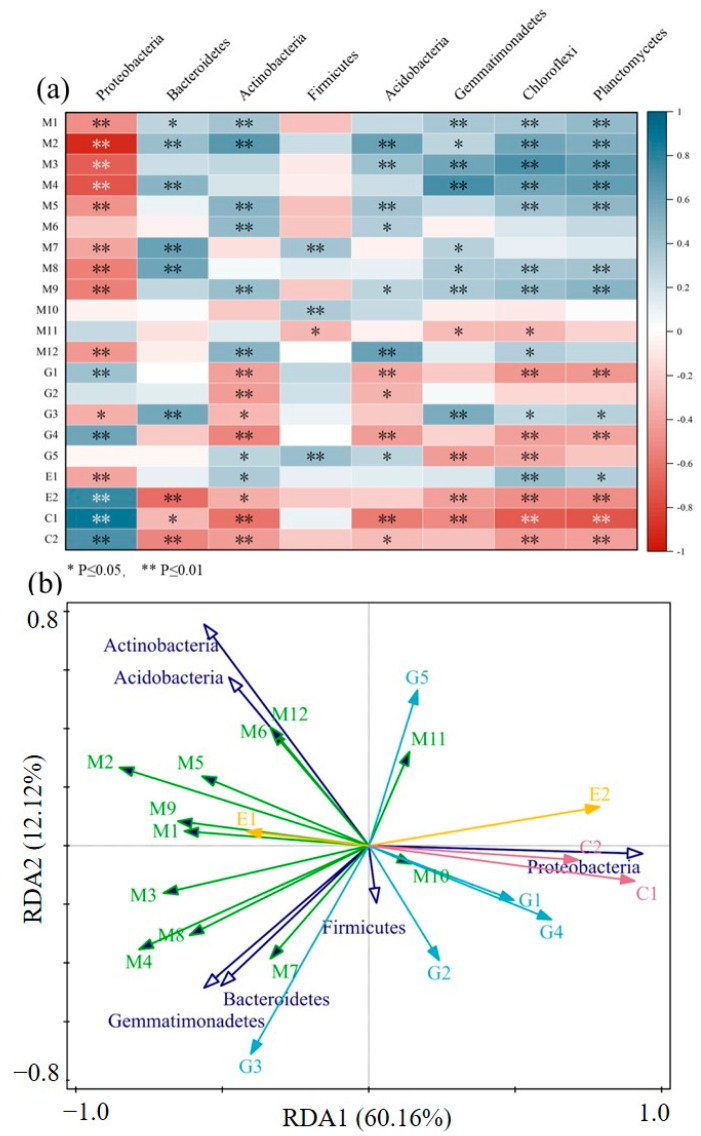
The Relationship between relative abundances of bacterial major secondary metabolic pathways and bacterial phyla (**a**): correlation analysis; (**b**): RDA analysis. M1: amino acid metabolism; M2: carbohydrate metabolism; M3: energy metabolism; M4: metabolism of cofactors and vitamins; M5: lipid metabolism; M6: xenobiotics biodegradation and metabolism; M: nucleotide metabolism; M8: metabolism; M9: metabolism of terpenoids and polyketides; M10: glycan biosynthesis and metabolism; M11: metabolism of other amino acids; M12: biosynthesis of other secondary metabolites; G1: replication and repair; G2: translation; G3: genetic information processing; G4: folding, sorting, and degradation; G5: transcription; E1: membrane transport; E2: signal transduction; C1: cellular processes and signaling; C2: cell motility.

A Pearson correlation analysis of the relative abundance for major sub-functions in the 2-level predicted functional layer of soil bacteria with the relative abundance of bacterial phylum was conducted, and a correlation heat map was drawn (Figure 4a). Proteobacteria, Actinobacteria, Acidobacteria, Gemmatimonadetes, Chloroflexi, and Planctomycetes are significantly correlated with the sub-functions of a bacterial 2-level predicted functional layer. Among them, metabolism pathways and membrane transport pathways were significantly negatively correlated with Actinobacteria, while they were significantly positively correlated with Acidobacteria, Gemmatimonadetes, Chloroflexi, and Planctomycetes. In contrast, genetic information processing pathways, such as replication and repair, translation, cellular processes, and information transfer and signaling had significant positive correlations with Actinobacteria and negative correlations with Acidobacteria, Gemmatimonadetes, Chloroflexi, and Planctomycetes, which contrasts with metabolism pathways and membrane transport pathways.

The main 2-level of bacteria sub-functions and bacterial phylum-level species were analyzed by RDA to understand the relationship between the main predicted functional pathways of soil bacteria with bacterial community structure in the study area (Figure 4b). The results of the analysis showed that the first and second axis explained 60.16% and 18.12% of the variations in a bacterial functional structure, respectively (F = 8.4, *p* = 0.002), with Proteobacteria (*p* = 0.002), Actinobacteria (*p* = 0.004), Firmicutes (*p* = 0.03), and Acidobacteria (*p* = 0.03) significantly affecting the functional structure of bacteria. The Proteobacteria accounted for 53.0% of the variation in the functional structure of bacteria and was the dominant species causing the difference in the functional structure of bacterial.

### 3.5. Characteristics of Soil CO_2_ Emissions during the Succession of Halophyte Vegetation

**Figure 5:**
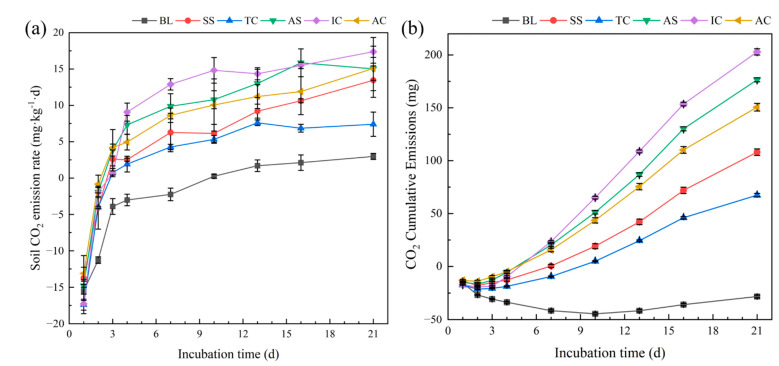
Variation of soil CO_2_ emissions with incubation time in various plots during the succession of halophyte vegetation in the Yellow River Delta. (**a**): CO_2_ emission rate; (**b**): CO_2_ cumulative missions.

Soil CO_2_ emission rates during the succession of halophyte vegetation in the study area were basically consistent over time (Figure 5a), with negative and non-significant differences in soil CO_2_ emission rates for each plot on the first day of incubation. The rate of soil CO_2_ emission in each plot increased significantly with time, with a faster rate of increase on the first four days and a slower rate of increase from the seventh day onwards and remained stable thereafter. The soil CO_2_ emission rate of the soil covered with vegetation all became positive on the third day, while the soil CO_2_ emission rate of the bare land changed from negative to positive on the 10th day, and the emission rate was always lower than that of the soil covered with vegetation.

The cumulative CO_2_ emissions for each plot during the incubation period are shown in Figure 5b. On the whole, only the CO_2_ emission fluxes of the bare land were negative during the incubation period, showing a sink for atmospheric CO_2_, while soils with vegetation cover, such as SS, TC and AS, showed a steady upward trend in cumulative emissions as a source of CO_2_. After 21 days of incubation, the amount of CO_2_ released by IC was the largest, reaching 202.87 mg, followed by AS, AC, and SS, with CO_2_ releases of 176.53 mg, 150.62 mg, and 108.06 mg, respectively. TC soil had a smaller CO_2_ release of 67.42 mg. The bare land showed CO_2_ absorption, and the cumulative absorption peak appears on the 10th day, which was 44.59 mg, the cumulative absorption was 28.29 mg after 21 days of incubation.

### 3.6. The Main Factors Influencing the Rate of Soil CO_2_ Emissions

**Figure 6:**
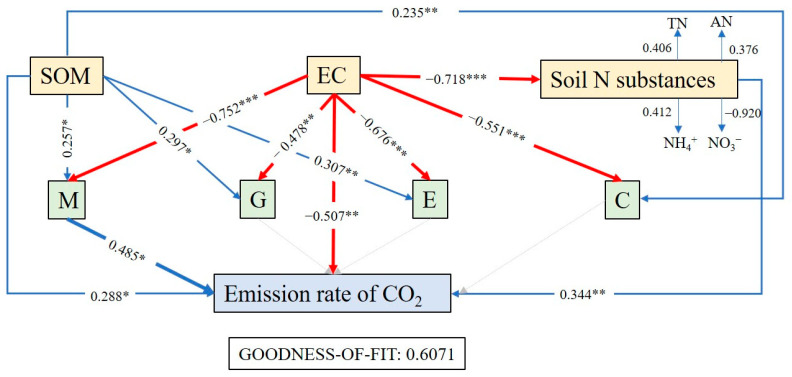
Correlation between soil CO_2_ emission rate and bacterial function prediction pathway and soil environmental factors. Notes: Coefficients that differ significantly are indicated by * *p* ≤ 0.05, ** *p* ≤ 0.01, or *** *p* ≤ 0.001. SOM stands for soil organic matter; EC stands for soil conductivity; Soil N substances stands for soil total nitrogen, AN stands for soil alkaline hydrolyzable nitrogen, NH_4_^+^ and NO_3_^−^; M stands for metabolism; G stands for genetic information processing; E stands for environmental information processing; C stands for cellular processes.

Partial least squares regression analysis of the mean CO_2_ emission rate of soils in the study area at each site during the incubation phase with the predicted functional pathway functional gene abundance of soil bacteria and soil environmental factors (Figure 6) showed that salinity was the primary factor affecting the predicted functional abundance and CO_2_ emission rate of the soil in the study area, with salinity showing a significant negative correlation with both functional gene abundance and CO_2_ emission rate. Furthermore, organic matter and soil nitrogen substances contributed significantly to the rate of soil CO_2_ emission in the study area and were also important factors affecting soil CO_2_ emission. Organic matter contributed significantly to the functional genes in the study area, but the effect of soil nitrogen substances on the abundance of functional genes was not significant. Only the abundance of metabolism functional genes showed a significant positive correlation with CO_2_ emission, which confirmed the central position of metabolism in the overall ecological function of bacteria.

## 4. Discussion

### 4.1. The Process of Halophyte Vegetation Succession Improves Soil Quality

In this study, it was found that the process of soil desalination in the Yellow River Delta soils was accompanied by an active succession of halophytes. Compared to the bare land at the beginning of the succession, soil salinity (conductivity) of SS, TC, AS, IC, and AC decreased significantly and organic matter and alkaline hydrolyzed nitrogen content increased significantly, organic matter can build up on the soil surface due to the biomass in the vegetated soil, leaf litter, and root activity [53]. The organic matter content of IC and AC was lower than that of AS, probably since IC and AC grow faster than other halophyte vegetation and consumed more nutrients, thus their organic matter content was not the highest among the sample plot in the study area.

Soil carbon and nitrogen accumulation varied markedly among the different halophyte vegetation, especially the alkali-hydrolyzable nitrogen content of AS and AC, which had more than twice that of the SS. This may be related to factors such as the type of soil litter and soil texture [54], where the type of litter, chemical composition, and decomposition of the litter affect the carbon and nitrogen pools of the soil and govern the physicochemical properties of the soil [55]. In general, the better the fertility of the soil, the higher the total and alkali-hydrolyzable nitrogen content of the soil. In this study, total and alkali-hydrolyzable nitrogen content of soils were significantly higher in lightly salt-tolerant vegetation (IC and AC) and moderately salt-tolerant vegetation (AS) than in the highly salt-tolerant vegetation (SS and TC). This indicates that the soil quality was improved during the succession of saline vegetation.

### 4.2. Halophyte Vegetation Succession Alters Bacterial Community Structure

In recent years, a large number of studies have investigated the effects of vegetation succession processes and changes in soil salinity gradients on microbial diversity, composition, and structure with mixed results. For instance, Zhang [56] found that vegetation type in loess hilly areas has an important influence on the structure and function of soil bacterial community composition, and Yang et al. [57] found a positive correlation between salinity and microbial diversity (Shannon, Chao1 index), while Wei et al. [58] found that the alpha-diversity and abundance of bacteria decreased with increasing salinity. The diversity of the soil bacterial community structure and the type of vegetation have long been thought to be closely related [59], and vegetation can have a direct or indirect impact on soil microorganisms through the inter-root environment and litter, which can offer a favorable living environment and nutrients for soil bacteria, as well as through the decomposition of apoplast and root secretions [16].

The results of this study showed that the OTU number and diversity as well as richness indices of soil bacteria increased during the succession of halophyte vegetation in the Yellow River Delta, and there were significant differences in the diversity index of soil bacteria between bare land and those with vegetation cover. Salinity was shown by Mantel tests to be the dominant factor causing changes in soil bacterial diversity, with high salinity increasing the extracellular osmotic pressure rate of bacteria [59,60], resulting in cell dehydration and inhibition or even death of bacteria that were not adapted to osmotic pressure, thus reducing bacterial diversity. The process of halophyte vegetation succession in the Yellow River Delta significantly affected the composition of bacterial community, with the highest abundance of Proteobacteria in each plot, accounting for more than half of the total abundance in the bacterial phylum, which is consistent with the findings of Guan et al. [61] and Ahmed al. [62] on bacterial communities in saline soils.

Proteobacteria can use atmospheric carbon and nitrogen sources to obtain energy for metabolism, which gives Proteobacteria dominance in saline soil [63]. Nevertheless, the relative abundance decreases during the succession of saline vegetation, mainly due to changes in the soil microenvironment and changes in the material-energy cycle during the succession of saline vegetation, and these changes inevitably affect the composition of the microbial community. Actinobacteria are mainly involved in the decomposition and metabolism of some macromolecular compounds, such as dead leaves, and the production of various antibiotics [64]. With the succession of halophytic vegetation, their abundance increased, probably due to the greater abundance of lightly saline sites with higher letter size, which contributed to the increase in the abundance of Actinobacteria, acidophilus, an acidophilic bacterium that is mainly concentrated in the inter-rhizosphere soil and has a high metabolic activity to degrade plant cellulose [65]. With the decrease of soil salinity during vegetation succession, the soil develops in a direction favorable to plant growth, the amount of vegetation increases, plant photosynthesis is enhanced, underground root secretions increase, and the abundance of Acidobacteria phylum increases. This is consistent with the findings of Yang Yunli [63] on soil bacteria during vegetation succession in the hilly areas of the Qianzhong Mountains. Both Bacteroidetes and Firmicutes had the highest abundance in the medium salt-tolerant vegetation TC sample site. Several studies have shown that Gemmatimonadetes mainly exist in saline soils and arid areas, and are relatively rare in soils, with relative abundance accounting for about 2% of the total bacterial community. Gemmatimonadetes accounted for 5.11–7.29% of the total in this study, demonstrating the high adaptability of the phylum to extreme environments. Phyllostomycetes can participate in a variety of ecological processes, grow slowly, do not compete with plants for soil nutrients, and have a low relative abundance in the soil bacterial community, consistent with the results of Sun Xin [66].

UPGMA cluster analysis showed a significant similarity in bacterial community structure in soils with the same degree of salt-tolerant vegetation, while soil bacterial community structure was more variable in soils with different degrees of salt-tolerant vegetation, probably due to soil environments with different salinity, affecting bacterial communities differently. Chowdhury et al. [67] showed that soil salinity affects the composition of soil microbial communities through osmotic potential. Rajaniemi and Allison [68] concluded that soil microbial community composition was more influenced by soil salinity than soil C and N factors, which is consistent with the findings of this work. The results of SIMPER analysis showed that *stenotrophomonas* is the bacterial group that contributes most to the variability of community structure, and it is the most dominant genus in the soil during vegetation succession in the Yellow River Delta, belonging to the class proteobacteria. Many genera under this class can survive not only through heterotrophic processes, but also through autotrophic processes to obtain energy for metabolism, have a great capacity for adaptation to adversity [69], and can survive by obtaining nitrogen sources through trace carbon sources and their nitrogen fixation.

### 4.3. Halophyte Vegetation Succession Alters the Functional Structure of Soil Bacteria

With the rapid development of bioinformatic function detection technology, PICRUSt function prediction can be achieved by constructing a “species-gene” relationship network, predicting gene type and abundance of the community by OTU, and comparing them with the KEGG database [36]. This study showed that PICRUSt function prediction was more reliable. Through PICRUSt function prediction, Lin Huiying et al. [70] found that the abundance of different functional genes showed regular changes with increasing altitude, and Zhang Tuo et al. [71] found that metabolic pathways were the core functions of soil bacteria in a study on different land use practices in the lower Songhua River wetlands. Soil bacteria utilize six types of ecological functions: metabolism, environmental information processing, genetic information processing, cellular processes, human diseases, and organic systems to maintain the stability of the ecosystem [72].

In this study, there was a diversity of soil bacterial functions in each plot of the study area, and the relative abundance of metabolism function genes was highest in each plot, indicating that metabolism is the most important function of bacteria in the study area, which is consistent with the findings of Zhang [71] and Liu [73]. The predicted functional abundance of soil was highly correlated with soil CO_2_ emission fluxes, indicating that the soil metabolic functional microbial groups are closely related to soil carbon cycling processes. During the succession of halophytic vegetation in the study area, the predicted functional abundance of soil bacteria gradually increased, indicating that with the succession of vegetation, the physiological activities of soil bacteria became more active, and their ability to utilize carbon sources such as amino acids and sugars became stronger. With the continuous cycling of ecosystem materials and energy, the natural succession of the vegetation inevitably leads to the functional refinement of soil bacteria [74].

Information on the abundance of functional genes in the 2-level predicted functional layer showed that the relative abundance of genes for amino acid metabolism, carbohydrate metabolism, energy metabolism, membrane transport, and replication and repair functional was high, with a total relative abundance of 43.48%, indicating that soil bacteria in the study area are mainly involved in amino acid, carbohydrate, energy metabolism, and material transport activities. The process of amino acid metabolism occurs through processes such as denitrification and transamination, converting amino acids into amines and keto acids and carbon dioxide, a process that is closely associated with the process of nitrogen cycling in soils and plants [74], and carbohydrate metabolism is closely related to carbon, nitrogen, and phosphorus cycling. Metabolism pathway function genes correlated significantly with nitrogen such as total, ammonia, and nitrate nitrogen and also showed a positive correlation with fast-acting phosphorus, indirectly indicating the role of metabolic function genes in the soil element cycle. A significant increase in the abundance of replication and repair functions during the succession of halophyte vegetation indicates accelerated bacterial growth and reproduction. Changes in the functional structure of microorganisms are closely related to structural changes [36,75], and with vigorous metabolism and growth of soil bacteria, the structural diversity of bacterial communities increases.

The ecological functions of soil bacteria are closely related to the composition of the bacterial community. Bacterial taxa such as Proteobacteria and Actinobacteria belong to the chemoenergetic heterotrophic group and play an essential role in carbon metabolism, organic matter decomposition, and elemental chemical cycling [76]. Predominant soil phylum in the study area was highly correlated with the predicted functional genes of the soil bacterial 2-level pathways, and the phylum Proteobacteria and Actinobacteria are the dominant bacterial phylum affecting the diversity of bacterial functional structures, and the centrality of the predicted functional metabolism described above was consistent with the strong metabolic functions of Proteobacteria and Actinobacteria reflecting the key role of these groups in the ecological functions of soil metabolism. A Mantel analysis showed that salinity, temperature, and nitrate nitrogen had significant effects on the soil 1-level predicted functional layer, with salinity being the key environmental factor affecting the structure of the soil bacterial community in the study area, as well as the key factor affecting the abundance of each predicted function of bacteria. High correlation of soil predicted functional genes with the dominant bacterial phylum in the soil illustrates the functional diversity of the bacterial phylum on the one hand, and the accuracy of PICRUSt functional annotation on the other.

### 4.4. Characteristics and Influencing Factors of Soil CO_2_ Emissions during the Succession of Halophyte Vegetation

Soil CO_2_ emissions are the most direct indicator to quantify the overall contribution of microbial participation in the carbon cycle process, and can reflect the ability of soil organic matter conversion and energy release [77], the accelerated rate of soil CO_2_ emissions represents an accelerated rate of soil material metabolism. In this study, the rate of soil CO_2_ emission gradually increased with positive succession of halophyte vegetation, which was consistent with the change in abundance of predicted functional genes at each site. Vegetation succession improves the soil environment and fertility, stimulates microbial decomposition activity and promoted soil respiration, and microorganisms are able to increase the diversity of nutrients used by plants, thus promoting plant growth [78].

In the first four days of incubation, the CO_2_ emission rate of each site was negative, which is because the incubation experiment in this study was under flooding conditions, and the soil microorganisms reduced CO_2_ in a relatively anaerobic environment, at which time the soil in the study area showed a sink of CO_2_. With the extension of the incubation time, the CO_2_ emission rate turned positive and became larger, and finally stabilized, indicating that the microorganisms were enriched during the incubation. In saline soils, salinity is the most important limiting factor on CO_2_ emission rate, and saline soils limit microbial activity due to soil infiltration pressure; soil organic matter is the material basis for microbial metabolic decomposition to release CO_2_, and microbial activity may also be limited by soil carbon sources due to low plant cover and differences in apoplastic quality in highly saline soils [79].

Under natural salinity gradients, soil CO_2_ emission rates were mostly significantly negatively correlated with salinity [7,67] and significantly positively correlated with carbon sources represented by organic matter [80], which is consistent with the results of this study. Soil CO_2_ emission rate pathway analysis showed that soil mineral nitrogen content showed a significant correlation with CO_2_ emission rate, suggesting that nitrogen content is also an important factor affecting microbial activity. Microbial life activities are controlled by nitrogen [81], which can alleviate the stress of soil salinity on microorganisms and thus affect the soil ecosystem carbon cycle. Both soil CO_2_ emission rate sets and soil CO_2_ emission rate with predicted functional layer showed a significant positive correlation between emission rate and abundance of metabolism-related functional genes, which also confirmed the above speculation that metabolic activity of soil bacteria is the core of microbial activity and metabolism plays an extremely important role in the succession of halophytic vegetation in the Yellow River Delta. The negative correlation between soil CO_2_ emission rates and the abundance of genes related to cellular activities is speculated to be due to the fact that most of the soil CO_2_ emissions originate from bacterial metabolic activities, which are much more active than various cellular processes such as cell growth and reproduction and cell movement.

## 5. Conclusions

(1) During the natural succession process of bare land—highly salt-tolerant vegetation—moderate salt-tolerant vegetation—lightly salt-tolerant vegetation, soil salinity continues to decrease, organic matter, total nitrogen, alkali-hydrolyzable nitrogen, and other nutrients continue to accumulate, and the succession of halophyte vegetation process is a process of continuous improvement of soil quality.
(2) Soil bacterial diversity and abundance increase during halophyte vegetation succession, with the phylum Proteobacteria being the most dominant phylum in the study area and *Stenotrophomonas* being the bacterial genus that contributes most to the variation in a soil bacterial community structure during salt vegetation succession. Bacteria in the study area are functionally diverse, with metabolism functions being their core functions.
(3) Soil CO_2_ emission rates increase continuously during the succession of halophyte vegetation in the Yellow River Delta. The CO_2_ emission rate of soils with vegetation cover is significantly higher than that of bare land. Under long-term flooding conditions, each site first becomes a sink of CO_2,_ and with longer flooding time each site changes from a sink to a source of CO_2_.
(4) Soil salinity is a major limiting factor for soil bacterial community structure, functional structure, and soil CO_2_ emission rates.

## Figures and Tables

**Table 1:** Physicochemical soil analysis of each plot in the Yellow River Delta.

Plots	BL	SS	TC	AS	IC	AC
T (°C)	18.41 ± 1.37 ^a^	17.32 ± 1.74 ^a, b^	16.07±1.75 ^b^	16.10 ± 1.49 ^b^	14.02 ± 1.05 ^c^	12.95 ± 2.89 ^c^
MC (%)	0.19 ± 0.02 ^a^	0.19 ± 0.03 ^a^	0.20 ± 0.02 ^a^	0.20 ± 0.02 ^a^	0.18 ± 0.02 ^a^	0.14 ± 0.04 ^b^
EC (dS·m^−1^)	4.83 ± 0.95 ^a^	3.27 ± 0.63 ^a, b^	2.89 ± 0.88 ^a, b^	2.27 ± 0.59 ^b^	0.24 ± 0.15 ^c^	0.39 ± 0.29 ^c^
pH	8.25 ± 0.42 ^a^	7.88 ± 0.37 ^b^	7.84 ± 0.27 ^b^	7.79 ± 0.26 ^b^	8.09 ± 0.23 ^a, b^	8.12 ± 0.23 ^a, b^
TN (g·kg^−1^)	0.22 ± 0.05 ^d^	0.32 ± 0.12 ^c, d^	0.26 ± 0.05 ^c, d^	0.57 ± 0.11 ^a^	0.49 ± 0.23 ^a, b^	0.41 ± 0.16 ^b, c^
SOM (g·kg^−1^)	5.87 ± 3.23 ^c^	11.40 ± 4.26 ^b^	12.98 ± 3.95 ^b^	17.06 ± 4.15 ^a^	14.88 ± 5.60 ^a, b^	12.39 ± 3.17 ^b^
AP (mg·kg^−1^)	2.24 ± 0.72 ^b^	3.86 ± 0.84 ^a^	2.66 ± 0.40 ^b^	2.85 ± 0.44 ^b^	2.31 ± 0.58 ^b^	2.84 ± 0.56 ^b^
AN (mg·kg^−1^)	12.30 ± 3.72 ^c^	18.62 ± 5.28 ^b, c^	27.14 ± 4.34 ^b^	43.58 ± 10.13 ^a^	40.95 ± 10.91 ^a, b^	36.05 ± 15.01 ^a, b^
NH_4_^+^ (mg·kg^−1^)	1.09 ± 0.31 ^b^	3.87 ± 1.24 ^a, b^	1.27 ± 0.58 ^b^	3.90 ± 0.98 ^a^	2.60 ± 0.82 ^a, b^	1.59 ± 0.54 ^b^
NO_3_^-^ (mg·kg^−1^)	2.64 ± 0.89 ^a, b^	3.20 ± 0.97 ^a, b^	3.71 ± 1.05 ^a^	3.24 ± 0.76 ^a, b^	2.48 ± 0.34 ^b^	2.25 ± 0.31 ^b^

Notes: Data in the table are “mean ± standard deviation”, and different lowercase letters in the same row represent significant differences between the indicators in different places (*p* < 0.05). T stands for temperature, MC stands for moisture content, EC stands for conductivity, TN stands for total nitrogen, SOM stands for soil organic matter, AP stands for available phosphorus, AN stands for alkali-hydrolyzable nitrogen, BL stands for the soil of bare land, SS stands for the soil of *Suaeda salsa* (L.) Pall., TC stands for the soil of *Tamarix chinensis* Lour., AS stands for the soil of *Aeluropus sinensis* (Debeaux) Tzvel., IC stands for the soil of *Imperata cylindrica* (L.) Beauv., AC stands for the soil of *Artemisia capillaris* Thunb., the same is represented below.

**Table 2:** The alpha diversity index of soil bacterial communities in the study area.

Plots	OTU	Shannon	Simpson	Chao1	ACE	Coverage
BL	2258 ^b^	5.83 ^b^	0.822 ^b^	3239 ^b^	3414 ^b^	0.979
SS	3339 ^a^	9.23 ^a^	0.990 ^a^	4387 ^a^	4489 ^a^	0.972
TC	3662 ^a^	9.26 ^a^	0.986 ^a^	5089 ^a^	5252 ^a^	0.967
AS	3697 ^a^	9.38 ^a^	0.989 ^a^	4909 ^a^	5029 ^a^	0.968
IC	3823 ^a^	9.92 ^a^	0.960 ^a^	5116 ^a^	5271 ^a^	0.971
AC	4134 ^a^	9.97 ^a^	0.993 ^a^	5389 ^a^	5384 ^a^	0.968

Notes: Different lowercase letters in the same column indicate significant differences between different plots for each indicator.

## Data Availability

The data will be available on request.

## Supplementary Materials

The following supporting information can be downloaded at: https://www.mdpi.com/article/10.3390/ijerph191912919/s1, Table S1: Contribution of major bacterial genera to spatial heterogeneity; Table S2: Absolute abundance table of genes for predicting the function of soil bacterial community in various plots of the Yellow River Delta (10^6^); Figure S1: Histogram of relative abundance of horizontal community structure of soil phyla in the Yellow River Delta; Figure S2: (a): Clade map of soil bacterial species in the Yellow River Delta; (b): UPGMA cluster analysis of soil bacteria in vegetation with different salt tolerance levels in the Yellow River Delta; Figure S3: Histogram of relative abundance of soil 1-level functional classification in the Yellow River Delta; Figure S4: Relative abundance of 2-level metabolic pathways in soil bacterial communities during the succession of halophyte vegetation in the Yellow River Delta.
