# Supplementary material for: Functional Diversity and CO2 Emission Characteristics of Soil Bacteria during the Succession of Halophyte Vegetation in the Yellow River Delta
Source: Int J Environ Res Public Health. 2022 Oct 9;19(19):12919. doi: 10.3390/ijerph191912919 (PMC9564505; doi:10.3390/ijerph191912919)
Supplement: Supplementary file 1 [file ijerph-19-12919-s001.zip › ijerph-1891470-supplementary.pdf]

Supplementary Materials

Table S1. Contribution of major bacterial genera to spatial heterogeneity.

Genera	Average Abundance(%)			
	**BL**	**H**	**Contribution (%)**	**Cumulative (%)**
Stenotrophomonas	49.52	9.60	36.67	36.67
Pseudomonas	4.21	0.54	3.844	40.51
Marinobacter	3.56	6.27	3.624	44.14
	G	M	Contribution (%)	Cumulative (%)
Stenotrophomonas	49.52	10.76	34.02	34.02
Pseudomonas	4.21	6.27	3.603	37.62
	G	L	Contribution (%)	Cumulative (%)
Stenotrophomonas	49.52	7.54	34.63	34.63
Acinetobacter	0.05	4.76	3.80	38.43
Pseudomonas	4.21	2.14	3.78	42.22
	H	M	Contribution (%)	Cumulative (%)
Stenotrophomonas	9.60	10.76	9.08	9.08
Marinobacter	6.27	1.64	5.292	14.37
Marinobacterium	-	-	2.20	16.57
	H	L	Contribution (%)	Cumulative (%)
Stenotrophomonas	9.60	7.54	6.36	6.36
Marinobacter	6.27	0.09	5.44	11.80
Acinetobacter	0.04	4.76	4.67	16.47
Lysobacter	-	-	2.09	18.56
	M	L	Contribution (%)	Cumulative (%)
Stenotrophomonas	10.76	7.54	7.67	7.67
Acinetobacter	0.03	4.76	4.83	12.50
Lysobacter	-	-	2.16	14.66
Marinobacterium	-	-	2.15	16.81

Notes: BL stands for the soil of bare land, H stands for the soil of highly salt-tolerant vegetation, M stands for the soil of moderate salt-tolerant vegetation, L stands for the soil of lightly salt-tolerant vegetation.

Table S2. Absolute abundance table of genes for predicting the function of soil bacterial community in various plots of the Yellow River Delta (10^6).

**1-Level**	**M**	**G**	**E**	**C**	**H**	**O**
BL	17.97	6.51	4.65	1.97	0.45	0.27
SS	20.88	6.87	5.31	1.87	0.45	0.33
TC	20.16	6.79	5.06	1.81	0.45	0.31
AS	21.57	7.08	5.45	1.95	0.45	0.34
IC	22.18	7.16	5.53	2.00	0.46	0.35
AC	23.88	7.62	5.93	2.09	0.49	0.37

**2-level**	**M1**	**M2**	**M3**	**M4**	**M5**	**M6**	**M7**	**M8**	**M9**	**M10**	**M11**	**M12**	**G1**	**G2**	**G3**	**G4**	**G5**	**E1**	**E2**	**C1**	**C2**	**C3**
BL	3.94	3.37	2.12	1.58	1.30	1.06	1.20	0.90	0.72	0.78	0.73	0.37	2.87	1.78	0.92	0.98	0.89	3.59	0.99	1.92	1.66	0.20
SS	4.55	4.00	2.44	1.82	1.59	1.37	1.36	1.06	0.90	0.84	0.80	0.39	2.98	1.92	1.07	1.02	0.95	4.29	0.95	1.67	1.50	0.25
TC	4.40	3.89	2.33	1.75	1.52	1.30	1.33	1.04	0.86	0.83	0.78	0.39	2.91	1.85	1.02	0.99	0.94	4.07	0.92	1.67	1.44	0.24
AS	4.66	4.18	2.54	1.88	1.63	1.39	1.40	1.07	0.92	0.87	0.82	0.42	3.07	1.97	1.08	1.05	0.99	4.40	0.98	1.68	1.56	0.26
IC	4.74	4.33	2.54	1.87	1.70	1.56	1.40	1.09	0.94	0.92	0.85	0.44	3.11	1.96	1.07	1.06	1.03	4.43	1.02	1.71	1.60	0.25
AC	5.11	4.71	2.73	2.00	1.83	1.69	1.50	1.15	1.02	0.95	0.92	0.49	3.32	2.07	1.13	1.12	1.12	4.76	1.08	1.75	1.65	0.27

M: Metabolism; G: Genetic Information Processing; E: Environmental Information Processing; C: Cellular Processes; H: Human Diseases; O: Organismal Systems. M1: Amino Acid Metabolism; M2: Carbohydrate Metabolism; M3: Energy Metabolism; M4: Metabolism of Cofactors and Vitamins; M5: Lipid Metabolism; M6: Xenobiotics Biodegradation and Metabolism; M: Nucleotide Metabolism; M8: Metabolism; M9: Metabolism of Terpenoids and Polyketides; M10: Glycan Biosynthesis and Metabolism; M11: Metabolism of Other Amino Acids; M12: Biosynthesis of Other Secondary Metabolites; G1: Replication and Repair; G2: Translation; G3: Genetic Information Processing; G4: Folding, Sorting and Degradation; G5: Transcription; E1: Membrane Transport; E2: Signal Transduction; C1: Cellular Processes and Signaling; C2: Cell_Motility; C3: Cell Growth and Death.

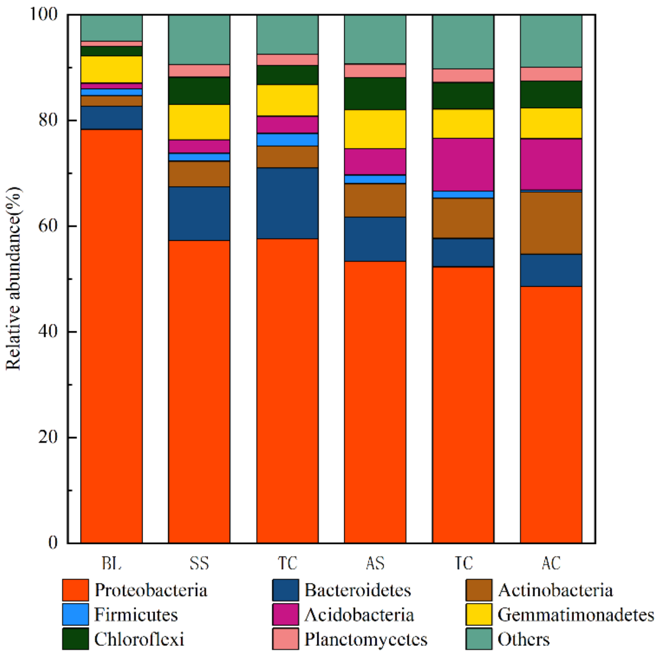

Figure S1. Histogram of relative abundance of horizontal community structure of soil phyla in the Yellow River Delta.

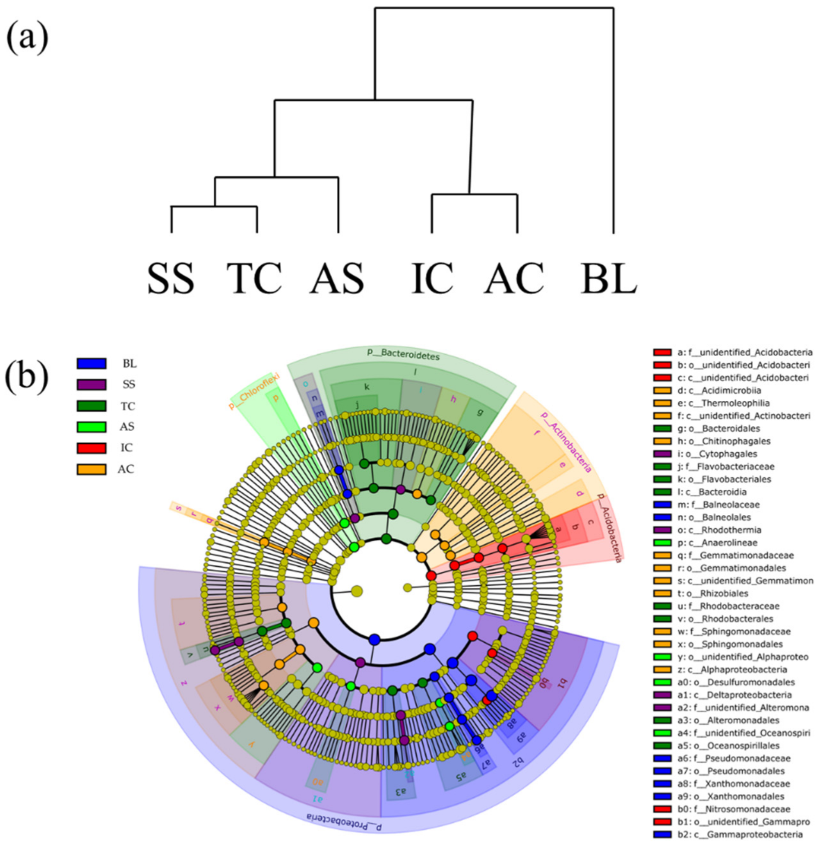

Figure S2. (**a**): Clade map of soil bacterial species in the Yellow River Delta; (**b**): UPGMA cluster analysis of soil bacteria in vegetation with different salt tolerance levels in the Yellow River Delta.

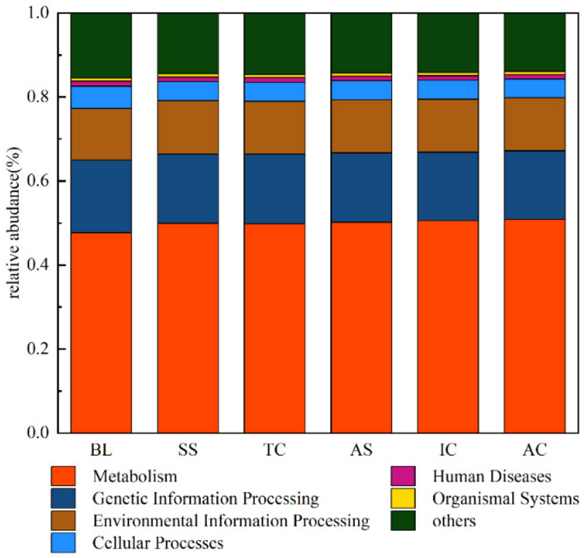

Figure S3. Histogram of relative abundance of soil 1-level functional classification in the Yellow River Delta.

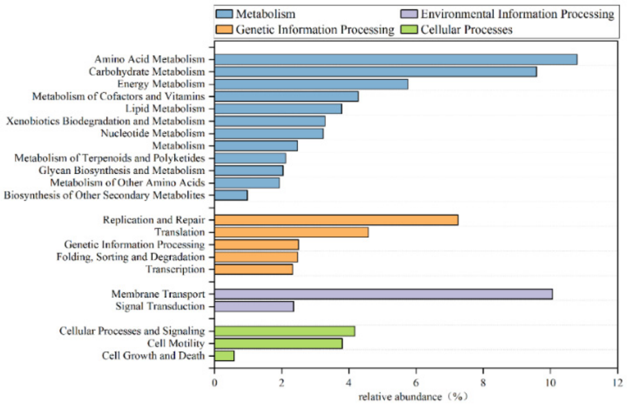

Figure S4. Relative abundance of 2-level metabolic pathways in soil bacterial communities during the succession of halophyte vegetation in the Yellow River Delta.
